# Mixed reality simulation for peripheral intravenous catheter placement training

**DOI:** 10.1186/s12909-022-03946-y

**Published:** 2022-12-17

**Authors:** Lauryn R. Rochlen, Elizabeth Putnam, Robert Levine, Alan R. Tait

**Affiliations:** 1 Department of Anesthesiology, University of Michigan, 1H247 University Hospital, 1500 E. Medical Center Drive, Ann Arbor, MI 48109 USA; 2 Emergency Care Center, Jackson Memorial Hospital, 1611 NW 12th Ave, Miami, FL 33136 USA; 3 ArchieMD, Inc., 6420 Congress Ave, Suite 2050, Boca Raton, FL 33487 USA; 4 Michigan Institute for Clinical & Health Research, 1600 Huron Parkway, Ann Arbor, MI 48105 USA

**Keywords:** Mixed-reality, IV placement, Peripheral intravenous catheter, Procedure training, Simulation

## Abstract

**Background:**

Despite the advantages of simulation-based training, trainees are typically unable to view internal anatomical structures. This limitation can be overcome by using mixed reality (MR) wherein 3-D virtual anatomical images can be projected. This study was designed to evaluate the efficacy of an MR trainer for peripheral intravenous catheter (PIVC) placement.

**Methods:**

Sixty-two participants used projected images of arm veins to place a PIVC in a mannequin arm. Participants were evaluated using a checklist on their ability to successfully place the PIVC. Participants completed a survey to elicit demographic information and perceptions of the trainer. A follow-up survey at two-weeks assessed clinical experiences with PIVC placement since using the MR trainer.

**Results:**

First attempt catheter placement was successful in 48 (77.4%) cases. Only 11 (17.7%) and 3 (4.8%) of participants caused ‘extravasation’ and ‘hematoma’ formation on their first attempt, respectively. Fifty-nine participants (95.2%) agreed that ability to see internal structures was useful, and 58 (93.5%, respectively) agreed that the interactivity promoted learning and that MR should be included in training.

**Conclusions:**

Results of this study showed that use of a novel MR trainer for PIVC placement appears to provide an environment conducive to successful learning. Most participants were successful at PIVC placement on their first attempt and an overwhelming number found it helpful in identifying landmarks and confirming correct needle angles for insertion. Given the increasing emphasis on simulation training, highly immersive MR tools appear to offer promise to close the gap between classroom instruction and clinical experience.

## Background

In recent years, there has been an increasing acceptance and adoption of the use of simulation as a means to enhance clinical procedural training. Use of simulators in medical education allows trainees to increase their exposure to a variety of medical procedures (particularly uncommon or rare procedures/conditions) in a safe environment in which mistakes have no patient consequences. This increased exposure to medical procedures through simulation is particularly important given concerns over expanding medical school classes and limited faculty availability [1, 2].

Although, simulation training has shown great promise there are several inherent disadvantages [3–6]. First, while simulation technology with mannequins provides the substrate (i.e., the simulated patient) with which to practice, it is limited by the inability of the trainee to visualize the internal anatomy or pathological states. Second, because few instruments used to evaluate procedural training skills have been validated, a lack of training fidelity may cause inconsistent training results and further, a lack of simulation faculty can be a barrier to effective procedural training [7–9]. A promising new approach to simulation training has been the introduction of virtual-reality (VR) and, more recently, mixed reality (MR) [10–15]. MR has advantages over VR in that it combines the virtual world with the real-world allowing 3-D images or animations to be displayed in the same view as real objects. Since the goal of an invasive medical procedure is to accurately and safely complete the procedure, the ability to identify anatomical landmarks is critical. In fact, modern medicine increasingly relies on some form of imaging (e.g., x-ray, ultrasound, or fluoroscopy) during invasive procedures. Traditional mannequins lack the ability to provide the trainee with an understanding of the body’s internal anatomy, and while they are effective at teaching psychomotor skills, they may have a limited capacity to convey critical teaching points such as the importance of positioning a patient in a certain manner as well as simulating complications that may occur, as a result of, or during a procedure. In the field of clinical simulation training, MR allows the trainee to identify internal anatomy and landmarks by super-imposing 3-D virtual anatomical images onto the surface of the mannequin. Studies have not only shown the benefits of MR in terms of acquisition of knowledge but also trainee preferences for its use [7, 16, 17].

With this in mind, this study was designed to develop and evaluate an MR program for procedural skills training. For the purposes of this study, peripheral IV catheter insertion was selected. This is a common procedure but has broad implications for a host of healthcare workers including physicians, nurses, phlebotomists, paramedics, and other medical personnel. The primary objective of this study was to assess the usability of the novel MR PIVC insertion.

## Methods

This study was deemed exempt from full review by the University of Michigan’s Institutional Review Board according to national regulations and therefore informed consent from participants was not required. This manuscript follows the modified STROBE guidelines for reporting the findings of observations studies in simulation education [18].

### Program development

**Fig. 1:**
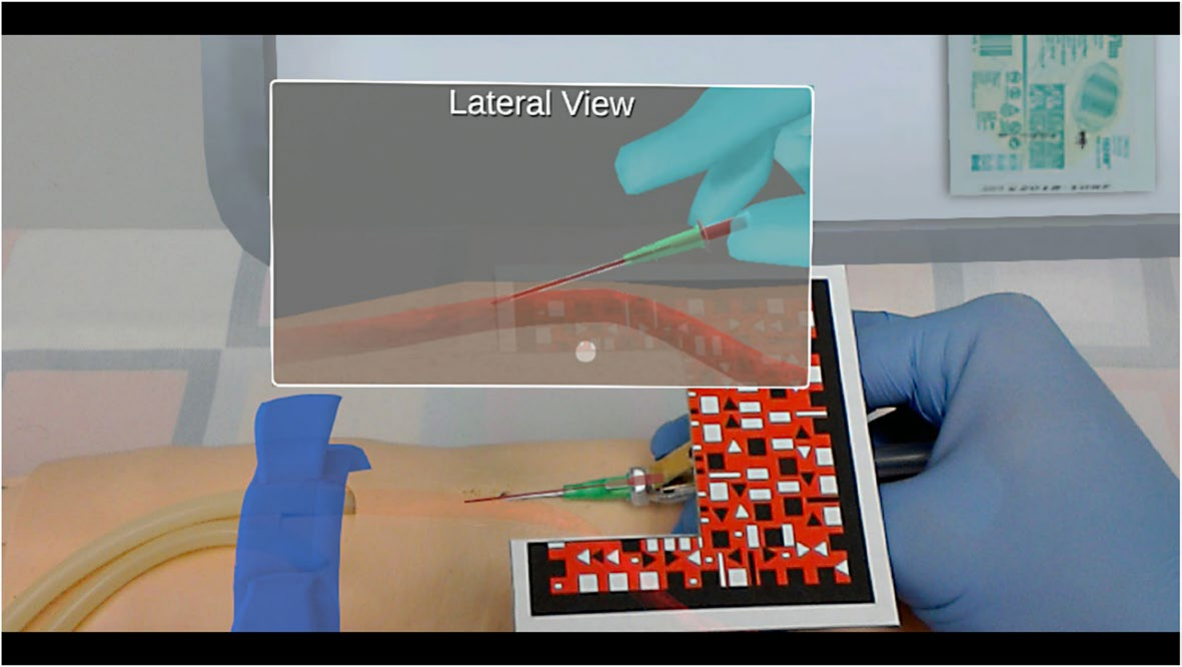
Image of participant using the mixed reality trainer to place a peripheral intravenous catheter

**Fig. 2:**
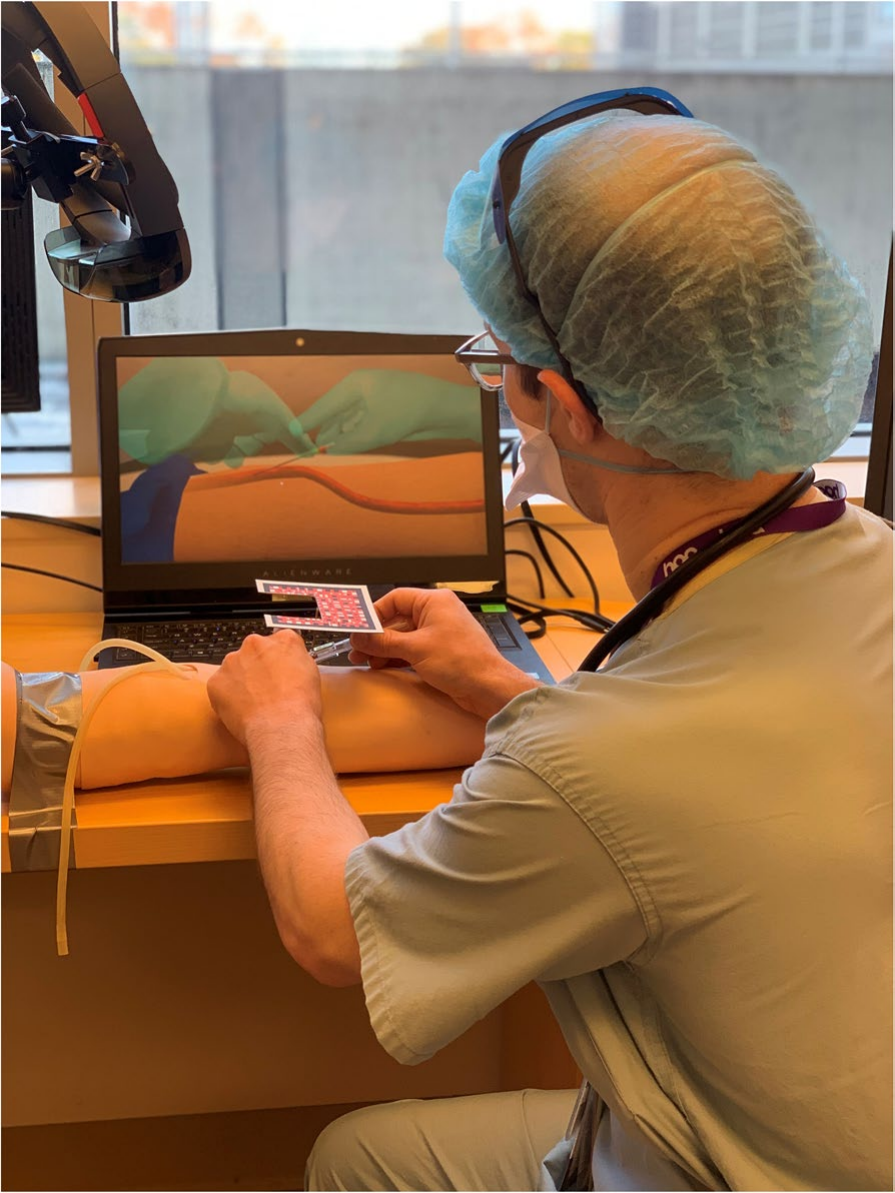
Image of set up showing HoloLens, screen, participant and mannequin arm

Content for the MR programs was defined by expert opinion (board certified emergency physician and anesthesiologists) using an iterative approach. Content scripts were defined, and mock-ups of the graphics reviewed and revised. Graphics were designed using both 2 and 3-D software. The principal graphic design programs utilized included Maya, 3D Studio Max, Adobe After Effects, Adobe Photoshop, and Macromedia Flash. Using these programs, models were developed and 3-D visuals rendered using the Unity game engine together with ArtToolKit. ArtToolKit is a powerful mixed reality application that allows for optical tracking e.g., movement of a catheter into a vein. Once the models were created, content was merged into procedure-specific interactive modules which, in concert with HoloLens technology (Microsoft Corp. Redmond WA), allows the user to view superimposed internal anatomical features on a mannequin via computer-generated images (Fig. 1). For the purposes of this program, the HoloLens was used to help the participants visualize the major arm veins superimposed on a Laerdal mannequin arm during catheter placement (Fig. 2).

### Usability testing

Once developed, the mixed reality program was tested for usability by 7 healthcare individuals with varying degrees of experience (none to experienced) with PIVC placement. Each individual attempted PIVC placement using the HoloLens and Laerdal arm mannequin. Individuals used a ‘talk back” approach to elicit feedback on their experiences with the mixed reality program. Comments were then relayed back to the development team for further review and refinement.

### Participants

The study population included medical students attending the University of Michigan’s Medical School together with residents, fellows, faculty, nurses, and nurse anesthetists (CRNAs) from the Departments of Anesthesiology and Pediatrics. Participants were identified through University of Michigan e-mail listings and face-to-face recruitment.

Several days prior to the start of the study, eligible participants were e-mailed a link to a short informational video of the MR technology and PIVC trainer. This video was also available to participants on the day of study who again had a chance to review and discuss with the investigators. Due to safety protocols related to the COVID-19 pandemic and minimization of disruptions to the MR calibrations, the participants did not wear the Hololens. The Hololens was secured to a stand over the mannequin arm and the participant was guided by the HoloLens projected images on to a monitor of the internal anatomy of the arm and arm veins where they would then attempt cannulation of the mannequin arm using a standard IV needle and catheter. This set-up can be seen in Fig. 2. During this procedure, participants were evaluated in real-time by one of the authors (LR, EP or AT) using a checklist approach for correct identification of the insertion site, angle of approach, and insertion and advancement of the needle and catheter. Successful placement of the PIVC on the first attempt was documented. Complications such as the development of a hematoma or extravasation were also documented together with the number of subsequent attempts required until successful placement. Following attempts at cannulation, participants completed a 28-item online survey (Qualtrics) to elicit information about their level of training, prior experience with PIVC placement, perceptions of the trainer and thoughts on the use of MR technology as a training tool for medical procedures in general. Survey items included both closed and open responses. Two weeks following the study, participants were e-mailed a link to a short 6-item online survey to elicit information about any experiences with PIVC placement in the clinical setting subsequent to their using the MR trainer and, its impact, if any, on their approach and success rates.

### Statistical analysis

Data from the Qualtrics surveys were downloaded directly to SPSS version 25.0 (IBM Inc., New York, NY) for analysis. Qualitative data were described using frequency distributions. Data are described as n (%) and mean ± SD. Free-text responses to open-ended questions were evaluated qualitatively and the most common themes reported.

## Results

Complete data were available for 62 participants. Table 1 describes the demographics of the participants. As shown, there was a wide range of experiences and training. Table 2 provides information on the knowledge base of the participants with respect to PIVC placement. Results showed that most participants were knowledgeable about the mechanics of PIVC placement and were aware of the potential complications.

**Table 1:**
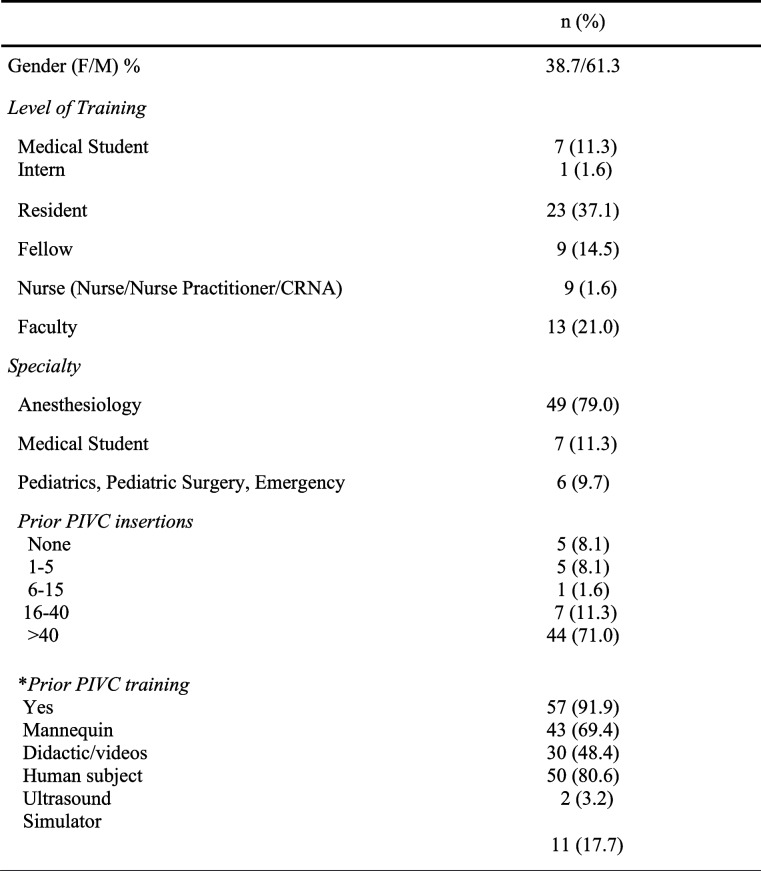
Participant demographics (*N* = 62)

**Table 2:** Knowledge of PIVC placement (*N* = 62)

	SD	D	Neither	A	SA
Know venous anatomy of arm	4 (6.6)	5 (8.2)	9 (14.8)	24 (39.3)	19 (31.1)
Familiar with equipment for PIVC	3 (4.9)	5 (8.2)	1 (1.6)	11 (18.0)	41 (66.1)
Knows proper procedure for PIVC	2 (3.3)	2 (3.3)	4 (6.6)	10 (16.4)	43 (70.5)
Knows techniques to verify placement	4 (6.6)	3 (4.9)	4 (6.6)	15 (24.6)	35 (57.4)
Knows common complications	4 (6.6)	3 (4.9)	4 (6.6)	18 (29.05	32 (52.5)

*SD* Strongly Disagree, *D* Disagree, *Neither* Neither disagree nor agree, *A* Agree, *SA* Strongly agree, Data are n (%)

Prior to using the MR trainer, participants were asked to rate their confidence in placing the PIVC. Results demonstrated a high overall confidence (8.40 ± 2.0 out of 10 where 10 = extremely confident).

The actions of the participants in placing the PIVC are described in Table 3, scored using a checklist approach. As shown, participants were very successful in following the correct procedures for catheter placement. First attempt catheter placement was successful in 48 (77.4%) of cases. Only 11 (17.7%) and 3 (4.8) of participants caused ‘extravasation’ and ‘hematoma’ formation on their first attempt.

**Table 3:** Participant checklist evaluation (*N* = 62)

	n (%)
Correctly place needle at insertion site	62 (100)
Hold needle at correct angle to skin	62 (100)
Advance needle	62 (100)
Lower the angle of insertion	62 (100)
Advance needle further into vein	60 (96.8)
Thread catheter and remove needle	59 (95.2)
*First attempt outcomes*	
Correct placement	48 (77.4)
Hematoma	3 (4.8)
Extravasation	11 (17.7)
*Subsequent successful attempts*	
1	10 (16.1)
2	1 (1.6)

After 2 e-mail reminders, 59/62 (95.2%) participants responded to the two-week follow-up survey. The responses are shown in Table 4. As shown, a majority of participants reported that exposure to the MR trainer improved their overall experience with PIVC placement and, over three-quarters believed it to be a potentially important bridge to ultrasound use. The participants’ responses to the Qualtrics evaluation survey are described in Table 5. Results from this survey suggest an overwhelming interest in using MR technology as a tool for medical procedure training.

**Table 4:** Two- week follow-up evaluation (*N* = 59)

	n (%)
*#Numbe of PIVCs since using MR*	
None	11(18.6)
1–5	15 (25.4)
6–10	14 (23.7)
11–20	8 (13.6)
> 20	11 (18.6)
*Successful PIVC placements since using MR	
0–25%	0 (0.0)
26–50%	1 (1.7)
51–75%	8 (13.8)
76–100%	37 (63.8)
*PIVC Success compared with Pre-MR experience	
Worse	0 (0.0)
About the same	42 (71.2)
Better	7 (11.9)
*How confident with PIVC since using MR	
Less confident than before	0 (0.0)
About the same as before	45 (84.9)
More confident than before	7 (13.2)
*MR useful in improving overall PIVC placement experience*	
Strongly disagree	3 (5.1)
Disagree	8 (13.6)
Neither disagree nor agree	23 (39.0)
Agree	24 (40.7)
Strongly agree	1 (1.7)
*MR trainer good adjunct to ultrasound*	
Strongly disagree	0 (0.0)
Disagree	0 (0.0)
Neither disagree nor agree	9 (15.3
Agree	33 (55.9)
Strongly agree	17 (28.8)

^a^Based on the number of participants who performed at least one PIVC placement in the 2 weeks following experience with the MR trainer

**Table 5:** Participants’ Perceptions of the Mixed Reality (MR) Trainer

	SD	D	Neither	A	SA
MR anatomy was realistic	2 (3.2)	4 (6.5)	6 (9.7)	42 67.7)	8 (12.9)
Ability to identify landmarks useful	2 (3.2)	3 (4.8)	7 (11.3)	42 (67.7)	8 (12.9)
Ability to see internal structures useful	0 (0.0)	1 (1.6)	2 (3.2)	40 (64.5)	19 (30.6)
MR trainer was easy to use	0 (0.0)	4 (6.5)	7 (11.3)	32 (51.6)	19 (30.6)
MR trainer was enjoyable	0 (0.0)	2 (3.2)	7 (11.3)	24 38.7)	29 (46.8)
MR improved ability to place IV	2 (3.2)	5 (8.1)	18 (29.0)	25 (40.3)	12 (19.4)
MR features support learning	0 (0.0)	1 (1.6)	4 (6.5)	37 (59.7)	20 (32.3)
MR interactivity promotes learning	0 (0.0)	0 (0.0)	4 (6.5)	36 (58.1)	22 (35.5)
MR novelty promotes learning	0 (0.0)	1 (1.6)	7 (11.3)	32 (51.6)	22 (35.5)
MR useful tool for skills training	1 (1.6)	0 (0.0)	12 (19.4)	26 (41.9)	23 (37.1)
Include MR in medical training	0 (0.0)	0 (0.0)	4 (6.5)	30 (48.4)	28 (45.2)
MR useful bridge to ultrasound	0 (0.0)	2 (3.2)	5 (8.1)	29 (46.8)	26 (41.9)

*SD* Strongly Disagree, *D* Disagree, *Neither* Neither disagree nor agree, *A* Agree, *SA* Strongly agree, Data are n (%)

Open-ended comments from participants regarding their perceptions of the MR trainer were overall very positive. A majority of these comments were related to the ability to see the internal anatomy, accurately judge the angle of needle placement and advancement, and the real-time feedback. The following comments were taken verbatim.

*“The level of detail of the anatomy of the venous system and the responsiveness from the MR when changing needle angles.”*

*“Good feedback/real time depiction of how micro movements can impact needle tip position.”*

*“The ability to measure angles and correlate visually, as it can be difficult to blindly make small angles.”*

*“Realistic feel, hands on opportunity without the pressure of hurting/missing on a patient.”*

Dislikes were minimal but focused primarily on the positioning of the QR code card on top of the needle which sometimes affected the way the needle could be held comfortably. It is expected that further iterations of this technology would mitigate this concern.

*“It was slightly difficult to hold the IV angiocath with that QR code attached.*

*“It was a little glitchy on my experience. I was not able to hold the needle as I would in real life given the placement of the marker, which detracted from the applicability of the device to a real- world situation. I also recommend wearing gloves during the simulation to make the experience more realistic (often we med students practice without gloves and it becomes very hard to perform the same steps with them on).”*

*“Doesn’t recreate the hardest part about IV placement which is how to handle the needle being in the vein while the catheter is still out of the vein.”*

*“Would be nice to eventually use the goggles and do the whole procedure with MR.”*

Participants were also asked to consider any potential barriers to implementation of the MR technology into clinical practice. A sample of the comments included the following:

*“Good to introduce early in training. Might be difficult to get some practitioners with lots of experience for something they feel they already mastered”.*

*“Resistance to technology among some team members as with any innovation. As they say, not all people like change.”*

*“It may encourage too much focus on the screen rather than actual device position, making translation to practice tenuous.”*

*“Cost”.*

Following use of the MR trainer, participants were asked to compare it with other forms of training that they had received in the past. Results showed that 39 (62.9%) found the MR technology to be superior/far superior to other procedural skills training methods. Only 3 (4.8%) stated that the MR technology was worse than other methods. Having experienced the MR trainer, participants were also asked how they would prefer to receive PIVC training in the future. While 12 (19.4%) reported that they would prefer training solely with the MR trainer, 32 (51.6%) reported that they would prefer a combination of instructional videos together with the MR trainer, and 13 (21.0%) would prefer videos, didactics, and the MR trainer. This suggests that a combination of videos and the MR trainer with or without didactic lectures was deemed the preferred method of instruction for PIVC placement.

## Discussion

Learning procedural skills is an essential part of most forms of medical training. While didactic instruction offers base level training, the majority of learning should take place in hands-on practice. Simulated practice is ideal for initial attempts prior to progressing to performing the procedures on patients. This MR module of peripheral IV catheter placement, therefore, offers a promising approach to enhance the current training capabilities for this procedure.

Traditional curriculum for IV placement for most medical professionals typically involves practice using task trainers and often, attempts on fellow learners. Benefits to MR in medical training for a variety of procedures and scenarios are now widely known [19, 20]. Advantages include the ability to reproduce the same scenario multiple times, accessibility, and realistic anatomy. Specific advantages to this PIVC module may be found in the ability to view internal anatomy while the procedure is being undertaken. This unique advantage provides the trainee with an appreciation of the three-dimensional anatomy and the potential complications that can occur. The majority of participants were able to achieve successful vein cannulation on the first attempt. In a similar anatomical procedural study looking at use of an MR platform, Schoeb et al. were able to show non-inferiority of an MR curriculum in training students in the placement of bladder catheters [21]. Furthermore, Moro et al. showed promise in VR and AR modules for teaching anatomy to students and showed that these modules are at least non-inferior to learning anatomy on only a tablet [22].

Encouragingly, even this brief interaction with the MR training module resulted in improved success in subsequent PIVC placements in the clinical settings. Across all levels of expertise, not only was confidence high, but additionally subsequent real-life PIV placements were successful. This suggests that there is skill acquisition, and it exemplifies an opportunity to engage learners in a practical application of their adult learning cycle. It is entirely possible that success and confidence in the clinical setting would continue to improve with repeated exposure to training with the MR module.

While there is certainly an underlying concern regarding the cost of simulation-based instruction and specifically MR platforms, the way this study was set up would allow for minimal expenses [23]. The set-up of a single HoloLens projetcing onto a screen rather than worn as a headset was a limitation imposed by Covid-19 protocols, but may be an advantage. One HoloLens provided many learners with the opportunity to interact with the MR module with the ease and reliabilty, unique advantages to using the Hololens in this fashion as opposed to having each learner wear the head mounted device.

The use of ultrasound guided IV placement is expanding [24, 25]. Practitioners have learned to appreciate the ability to view the internal anatomy to help guide the needle and catheter placement. As shown in the results from the follow-up survey, there was considerable support for use of the MR training module and a majority of participants believed l it could be a useful training bridge, as it specifically demonstrates the anatomy, and enables learners to view and recognize the significance of placing both needle and catheter within the target vessel.

Limitations of this study include that it was conducted at a single institution, although a variety of medical specialties and grades were recruited. A larger study population may yield more detail. Although some participants mentioned technical glitches, and the disadvantage of using a screen instead of the HolenLens headset for the research, the prototype nature of the MR module was recognized.

This study supports a novel way to use MR technology to teach the detailed anatomy and complications as well as the procedural skills required for successful peripheral intravenous catheter placement. Training for this procedure has broad connections to many medical disciplines and should be considered a welcome addition for learners.

## Data Availability

(data transparency): Participant data is available via Qualtrics. The datasets used and/or analysed during the current study available from the corresponding author on reasonable request.
